# Alpha-fetoprotein kinetics in patients with hepatocellular carcinoma receiving ramucirumab or placebo: an analysis of the phase 3 REACH study

**DOI:** 10.1038/s41416-018-0103-0

**Published:** 2018-05-29

**Authors:** Ian Chau, Joon Oh Park, Baek-Yeol Ryoo, Chia-Jui Yen, Ronnie Poon, Davide Pastorelli, Jean-Frédéric Blanc, Masatoshi Kudo, Tulio Pfiffer, Etsuro Hatano, Hyun Cheol Chung, Katerina Kopeckova, Jean-Marc Phelip, Giovanni Brandi, Shinichi Ohkawa, Chung-Pin Li, Takuji Okusaka, Yanzhi Hsu, Paolo B. Abada, Andrew X. Zhu

**Affiliations:** 1 Department of Medicine, Royal Marsden Hospital, Sutton, Surrey, SM2 5PT UK; 2 Division of Hematology-Oncology, Department of Medicine, Samsung Medical Center, Sungkyunkwan University School of Medicine, Seoul, 135-710 Korea; 3 Department of Oncology, Asan Medical Center, University of Ulsan College of Medicine, Seoul, 05505 Korea; 4 Department of Internal Medicine, National Cheng Kung University Hospital, College of Medicine, National Cheng Kung University, Tainan, 701 Taiwan; 5 Departmentof Surgery, The University of Hong Kong, Pokfulam, Hong Kong; 6 Department of Oncology, Santa Maria del Prato Hospital, Feltre (Belluno), 32032 Italy; 7 Department of Hepato-Gastroenterology and Medical Oncology, CHU de Bordeaux, Hôpital Haut-Lévêque, 33604 Pessac, France; 8 Department of Gastroenterology and Hepatology, Kindai University Faculty of Medicine, Osaka-Sayama, 589-8511 Japan; 9 Department of Medical Oncology, Instituto do Câncer do Estado de São Paulo, São Paulo, 01246-000 Brazil; 10 Department of Surgery, Graduate School of Medicine, Kyoto University, Kyoto, 606-8507 Japan; 11 Department of Medical Oncology, Yonsei Cancer Center, Yonsei University College of Medicine, Seoul, 03722 Korea; 12 Department of Oncology, University Hospital Motol, 2nd Faculty of Medicine of Charles University, 150 00 Praha, Czech Republic; 13 Department of Gastroenterology and Digestive Oncology, University Hospital of St Etienne, 42100 Saint Etienne, France; 14 Department of Experimental, Diagnostic and Specialty Medicine, University Hospital S. Orsola, 40138 Bologna, Italy; 15 Division of Hepatobiliary and Pancreatic Oncology, Kanagawa Cancer Center, Yokohama, 241-0815 Japan; 16 Division of Gastroenterology and Hepatology, Department of Medicine, Taipei Veterans General Hospital, Taipei, 112 Taiwan; 17 National Yang-Ming University School of Medicine, Taipei, 112 Taiwan; 18 Department of Hepatobiliary and Pancreatic Oncology, National Cancer Center Hospital, Tokyo, 104-0045 Japan; 19 Eli Lilly and Company, New York, NY 10016 USA; 20 Eli Lilly and Company, Indianapolis, IN 46285 USA; 21 Department of Medicine, Harvard Medical School, Massachusetts General Hospital, Boston, MA 02114 USA

**Keywords:** Hepatocellular carcinoma, Predictive markers

## Abstract

**Background:**

Post-hoc analyses of AFP response and progression and their relationship with objective measures of response and survival were performed in patients from REACH.

**Methods:**

Serum AFP was measured at baseline and every 3 cycles (2 weeks/cycle). Associations between AFP and radiographic progression and efficacy end points were analysed.

**Results:**

Median percent AFP increase from baseline was smaller in the ramucirumab than in the placebo arm throughout treatment. Time to AFP progression (HR 0.621; *P* < 0.0001) and to radiographic progression (HR 0.613; *P* < 0.0001) favoured ramucirumab. Association between AFP and radiographic progression was shown at 6 (OR 6.44, 95% CI 4.03, 10.29; *P* < 0.0001) and 12 weeks (OR 2.28, 95% CI 1.47, 3.53; *P* = 0.0002). AFP response was higher with ramucirumab compared with placebo (*P* < 0.0001). More patients in the ramucirumab arm experienced tumour shrinkage and AFP response compared with placebo. Survival was longer in patients with AFP response (13.6 months) than in patients without (6.2 months), irrespective of treatment (HR 0.457, *P* < 0.0001).

**Conclusions:**

Treatment with ramucirumab prolonged time to AFP progression, slowed AFP increase and was more likely to induce AFP response. Similar benefits in radiographic progression and response correlated with AFP changes.

## Introduction

Liver cancer is the sixth most commonly diagnosed cancer worldwide and the second most common cause of cancer death.^1^ Hepatocellular carcinoma (HCC) represents approximately 90% of primary liver cancers and occurs most frequently in patients with cirrhosis from chronic hepatitis B or C virus infection or alcohol abuse.^2^

Alpha-fetoprotein (AFP) level has long been known to correlate with HCC prognosis and has historically played a role in diagnosis. Elevated AFP levels are associated with larger tumours, bilobar involvement, portal vein invasion, poorly differentiated histology and decreased median survival.^3^ Measurement of AFP level has been incorporated into some HCC prognostic scoring systems.^4,5^ While high levels of AFP are recognised as a poor prognostic factor, the utility of AFP response or progression during anticancer treatment is still unclear. There are limited studies in patients with HCC correlating AFP kinetics with treatment response during locoregional therapy or while on sorafenib and no published results of patients on second-line treatment.

Vascular endothelial growth factor (VEGF) is overexpressed in HCC and associated with poorer clinical outcomes, suggesting VEGF-mediated signalling is important in HCC pathogenesis and is a therapeutic target.^6–8^ Ramucirumab is a recombinant immunoglobulin G, subclass 1 monoclonal antibody that specifically binds to the extracellular domain of VEGFR-2 with high affinity, preventing binding of VEGF ligands and receptor activation.^9^ REACH, a global, randomised, double-blinded placebo-controlled Phase 3 study, evaluated the efficacy and safety of single-agent ramucirumab for patients with advanced HCC after prior treatment with sorafenib (*N* = 565).^10^ Significant improvement in overall survival (OS) in the intent-to-treat (ITT) population was not achieved. However, a clinically meaningful improvement in OS was observed in patients with elevated baseline AFP levels (≥400 ng/mL [*n* = 250]) treated with ramucirumab vs placebo (OS 7.8 vs 4.2 months, respectively; hazard ratio (HR) 0.67, *P* = 0.006).

In the REACH study, AFP values were collected at baseline and during treatment. Post-hoc analyses of AFP response and progression, and correlations with other measures of efficacy including time to progression (TTP), objective response rate (ORR), and OS, were performed.

## Patients and methods

### Patient selection, randomisation and masking

The details of eligibility for inclusion in the REACH trial were previously described.^10^

### Procedures

Patients received either ramucirumab 8 mg/kg (ImClone Systems Corporation, Branchburg, NJ, USA) (*n* = 283) or placebo (*n* = 282) intravenously every 2 weeks until disease progression, unacceptable toxicity or withdrawal of consent. All patients received supportive care. Predefined dose modifications were allowed to manage treatment-related toxicity.^10^ Local radiological imaging was performed at baseline, every 6 weeks over the first 6 months of treatment and every 9 weeks thereafter. In the event of ramucirumab/placebo dose delays or missed doses, disease assessment and imaging studies were to be undertaken according to the original study schedule (i.e. every 6 weeks after first dose for the first 6 months and every 9 weeks thereafter), regardless of the actual number of on-study treatments received.

### Statistical definitions

TTP was defined as the time from randomisation to radiographic progression; radiographic response was assessed by protocol-defined criteria based on Response Evaluation Criteria in Solid Tumors 1.1 (appendix); ORR was defined as the proportion of patients who achieved complete response (CR) or partial response (PR) as their best overall response (BOR).

OS was defined as the time from randomisation to death from any cause.

Serum AFP levels were measured locally at baseline (within 2 weeks prior to randomisation), and every 3 cycles, i.e. every 6 weeks until treatment discontinuation, and at short-term follow-up. AFP progression was defined as ≥20% increase from non-zero baseline and absolute increase ≥10 ng/mL. For the small number of patients (*n* = 4; 2 in the ramucirumab arm, 2 in the placebo arm) with a true baseline AFP of zero, AFP progression was defined as absolute increase AFP ≥ 10 ng/mL from zero baseline. These definitions were chosen to limit the risk of non-significant variations in AFP levels being considered AFP progression. AFP response was assessed in the population subset with baseline AFP ≥ 1.5 upper limit of normal (ULN) and was defined as ≥20% decrease from baseline. This threshold of a minimum level of baseline AFP was selected to allow for a meaningful analysis since patients with very low levels of baseline AFP experiencing non-significant variations in AFP levels during treatment could result in large percent changes. Changes of 20 and 50% from baseline have been examined in previous studies.^11–13^

### Statistical analysis

This post hoc analysis was conducted within the ITT population of REACH. The baseline distribution of patients by AFP level was plotted for comparison between arms. After taking log10 of baseline AFP values, the frequency (patient count) was plotted for each arm. AFP response rate is presented with 95% confidence interval (CI) and was compared using the Cochran–Mantel–Haenszel test. Percent change in AFP from baseline was analysed for each arm at each time point up to Cycle 12. Analyses evaluated the association between the events of AFP progression and radiographic progression in each AFP level measurement time interval (Fisher’s exact test and odds ratio [OR]).

Time to AFP progression and time to radiographic progression between treatment arms were evaluated by the Kaplan–Meier method and tested by a stratified log-rank test. HR was generated using a stratified Cox proportional hazard model. AFP response rate is presented with 95% CI and compared using Cochran–Mantel–Haenszel test. The statistical analysis was done using the SAS® software Version 9.2.

AFP percent changes observed in patients in the ramucirumab arm were compared to those in the placebo arm at Cycles 3, 6, 9 and 12 by non-parametric Wilcoxon rank-sum tests.

## Results

**Fig. 1:**
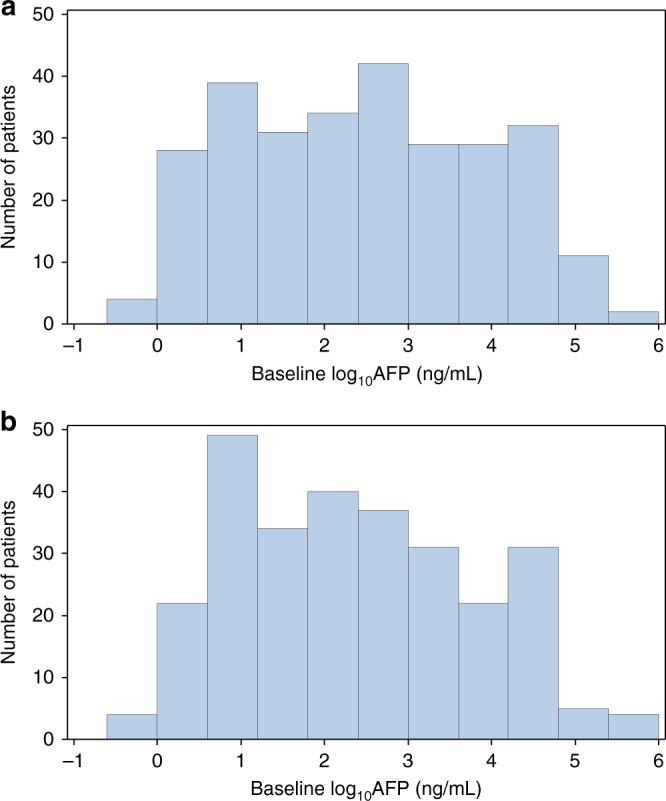
Distribution of patients by baseline AFP level in each arm. **a** Patients treated with placebo and best standard of care. **b** Patients treated with ramucirumab and best standard of care. AFP alpha-fetoprotein

Baseline patient and disease characteristics in the ITT population were well balanced between treatment groups.^10^ Baseline characteristics of patients in whom AFP response was assessed (with baseline AFP above 1.5 ULN, *n* = 417) were also well balanced and, apart from baseline, AFP showed no meaningful differences from the baseline characteristics of the ITT population (Table S1). Additional details on the REACH study population have been disclosed previously.^10^ After log transformation of baseline AFP, the distribution of patients by log AFP for each treatment arm appeared similar in both treatment arms. A *t*-test on the log-transformed baseline AFP levels did not show any significant difference in baseline AFP levels between two arms (Fig. 1).

**Fig. 2:**
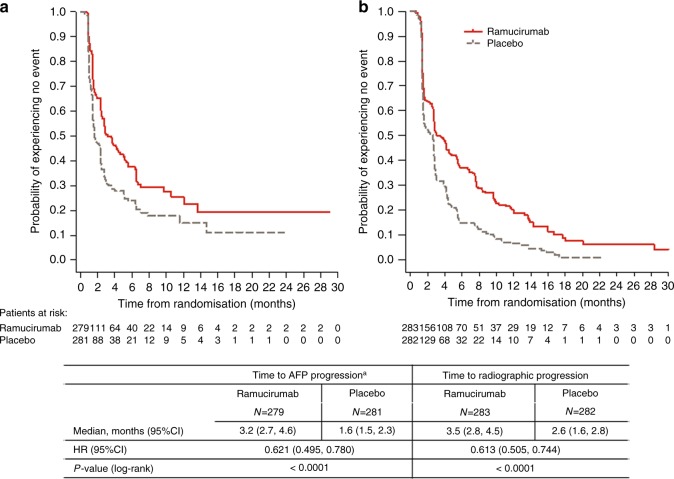
Kaplan–Meier plots of **a** time to AFP progression^a^ and **b** time to radiographic progression in the ITT population. AFP alpha-fetoprotein, CI confidence interval, HR hazard ratio, ITT intention to treat. ^a^Five patients were not included because of missing baseline AFP level

Changes in AFP relative to baseline were analysed and defined as either AFP progression or response, or neither, as per the Patients and methods section. A Kaplan–Meier plot of time to AFP progression for patients treated with ramucirumab vs placebo is shown in Fig. 2. The median time to AFP progression was 3.2 months in the ramucirumab arm (95% CI 2.7, 4.6, *n* = 279) and 1.6 months in the placebo arm (95% CI 1.5, 2.3, *n* = 281) with an HR of 0.621 (*P* < 0.0001).

Consistent with the results on time to AFP progression, patients treated with ramucirumab were more likely to experience an AFP response (decrease) at any time post-baseline compared to patients treated with placebo (ramucirumab: 27.8% vs placebo: 10.8%; *P* < 0.0001) and less likely to experience AFP progression (increase) at any time post-baseline compared to those treated with placebo (ramucirumab: 62.4% vs placebo: 75.9%; *P* = 0.0033). The difference in the percentage of patients with AFP response or progression between arms was significant when AFP response and progression were defined as a 20% (*P* < 0.0001) or 50% (*P* = 0.0004) change from baseline. No meaningful differences in the rates of AFP response were observed for patients with baseline ≥400 ng/mL compared to those with AFP < 400 ng/mL, suggesting that AFP response was independent of the magnitude of baseline AFP (data not shown).

**Fig. 3:**
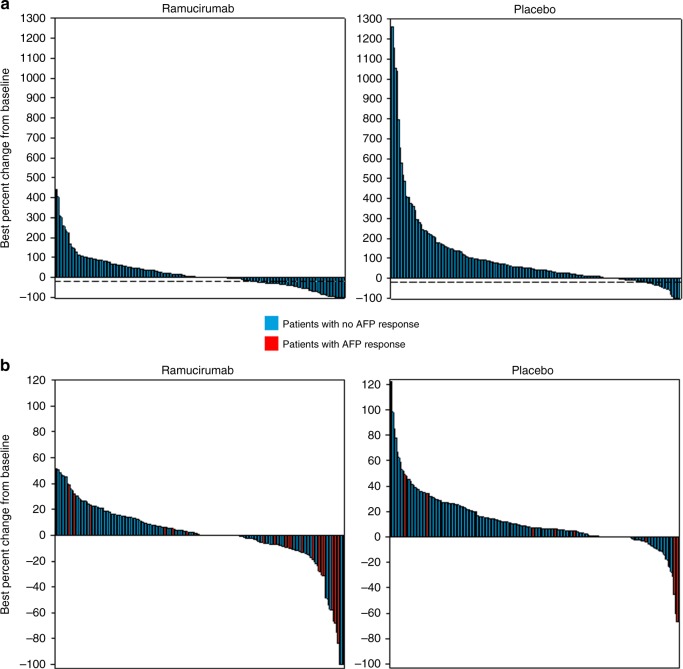
Waterfall plots of response for patients by treatment arm. **a** Best percent change in AFP from baseline measurements by treatment arm. **b** Best percent change in radiographic tumour response, and relationship with AFP response. AFP alpha fetoprotein

Waterfall plots of best percent change in AFP from baseline for patients treated with ramucirumab or placebo also support the results of the analyses on AFP response and progression (Fig. 3a). The proportion of patients who experienced an increase in AFP was not only lower in the ramucirumab arm but the magnitude of the increase also appeared smaller when compared with the placebo arm. Of note, 23 patients on the placebo arm also experienced an AFP response. An assessment of baseline characteristics for these patients did not identify any meaningful differences from the rest of the cohort, and other definitions of AFP response would not eliminate the presence of patients with AFP response in the placebo arm and likely represent true spontaneous responses.

**Fig. 4:**
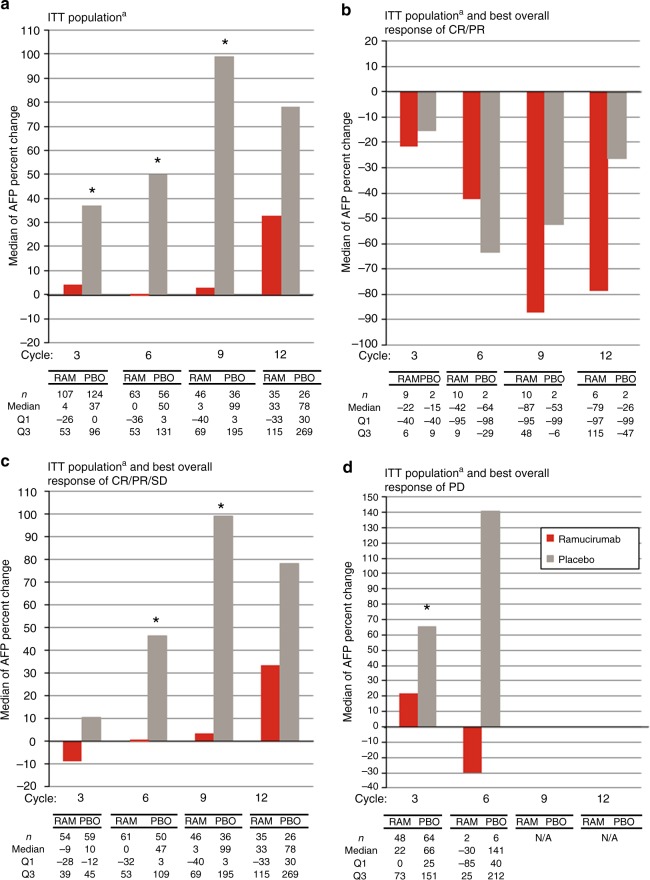
AFP percent change from baseline by cycle. Medians of AFP percent changes from baseline were plotted every three cycles for patients from the ITT population with baseline AFP ≥ 1.5 ULN by treatment arm. **a** for all patients; **b** for patients with best overall response of CR/PR; **c** for patients with best overall response of CR/PR/SD; **d** for patients with best overall response of PD. ^a^ITT population with baseline AFP ≥ 1.5 ULN. *Indicates a statistical difference between the two groups by non-parametric Wilcoxon rank-sum tests. AFP alpha-fetoprotein, CR complete response, ITT intention to treat, NA not available, PD progressive disease, PR partial response, SD stable disease, Q1 lower quartile, Q3 upper quartile

To further assess the kinetics of AFP during treatment, AFP percent changes from baseline were calculated, and the median percent change from baseline evaluated by treatment arm (Fig. 4a). At each AFP assessment time point following baseline at Cycles 3, 6, 9 and 12, the median percent increase in AFP level from baseline was smaller in the ramucirumab arm (4, 0, 3, 33%) than in the placebo arm (37, 50, 99, 78%), respectively, and AFP percent change was significantly smaller in the ramucirumab arm at Cycles 3, 6 and 9.

### Correlation of AFP changes with measures of radiographic response or progression

Kaplan–Meier plots of time to AFP progression and time to radiographic progression were similar in appearance (Fig. 2). The median time to radiographic progression was 3.5 months on the ramucirumab arm (95% CI 2.8, 4.5, *n* = 283) and 2.6 months in the placebo arm (95% CI 1.6, 2.8, *n* = 282, HR 0.613, *P* < 0.0001). A high association between AFP progression and radiographic progression occurring within each tumour assessment period was also observed (OR 6.4, 95% CI 4.0, 10.3, *P* < 0.0001 for up to Week 6, OR 2.3, 95% CI 1.5, 3.5, *P* = 0.0002 for Weeks 6–12) (Table 1).

**Table 1:** Radiographic progression and AFP progression by tumour measurement period

	Radiographic progression event	No radiographic progression event	*P*-value	Odds ratio (95% CI)
Up to 6 weeks, *N*	97	463		
AFP progression, *n* (%)	56 (58)	81 (18)		
No AFP progression, *n* (%)	41 (42)	382 (83)		
			<0.0001	6.4 (4.0, 10.3)
6–12 weeks, *N*	159	246		
AFP progression, *n* (%)	63 (40)	55 (22)		
No AFP progression, *n* (%)	96 (60)	191 (78)		
			0.0002	2.3 (1.5, 3.5)

*AFP* alpha-fetoprotein, *CI* confidence interval

AFP progression was defined as ≥20% increase from non-zero baseline and absolute increase ≥10 ng/mL, or absolute increase AFP ≥ 10 ng/mL from zero baseline, within 2 weeks

Median percent change in AFP was further assessed in subgroups of patients defined by their best overall radiographic response (objective response [complete response/partial response (CR/PR)], disease control [CR/PR/stable disease (SD)] and progressive disease [PD]). For patients with a best overall radiographic response of CR/PR, the observed median percent change in AFP was a decrease in both treatment arms, with more patients in the ramucirumab arm experiencing an objective response compared to placebo (Fig. 4b). However, this should to be interpreted with caution given the small number of patients with best response of CR/PR and the differences between the groups were not statistically significant (Fig. 4b). For patients with a BOR of disease control (CR/PR/SD), the median percent AFP increase from baseline for patients in the ramucirumab arm was lower than what was observed in the placebo arm at each cycle (Fig. 4c), with AFP percent changes being statistically different for the two arms at Cycles 6 and 9. In patients experiencing a best response of PD defined by radiographic progression, AFP increase from baseline for patients on the ramucirumab arm was significantly lower than what was observed on the placebo arm at Cycle 3 (Fig. 4d). There was no data available at Cycle 9 or 12 for this subgroup of patients as most patients with a best response of progression had already discontinued treatment.

Waterfall plots of radiographic tumour response by treatment arm and the relationship with AFP response (yes vs no) are shown in Fig. 3b. A higher proportion of patients experienced a radiographic response in the ramucirumab arm compared with the placebo arm. Most patients with a radiographic response (14 on RAM, 4 on PBO) also experienced an AFP response (10 on RAM, 3 on PBO).

### Overall survival by AFP response

**Fig. 5:**
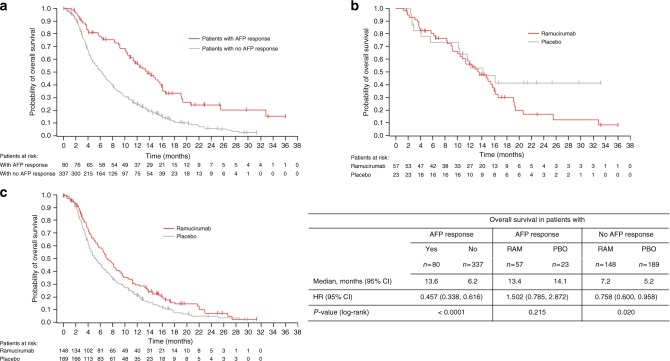
Kaplan–Meier plots of overall survival. **a** By AFP response, both arms combined. **b** By arm, in patients with AFP response. **c** By arm, in patients with no AFP response. AFP alpha-fetoprotein, CI confidence interval, HR hazard ratio, RAM ramucirumab, PBO placebo

Additional analyses on the relationship between AFP response and OS were performed. A Kaplan–Meier plot of OS for patients (baseline AFP > 1.5 × ULN), irrespective of treatment arm, with either an AFP response (*n* = 80) or no AFP response (*n* = 337) is shown in Fig. 5a. The median OS for patients with an AFP response was significantly longer than that for patients without AFP response (13.6 vs 6.2 months, HR = 0.457, 95% CI 0.338, .616; *P* < 0.0001).

Kaplan–Meier plots of OS by treatment arm in patients with either an AFP response (Fig. 5b) or no AFP response (Fig. 5c) are shown in Fig. 5. In patients with an AFP response, there was no statistically significant survival benefit of ramucirumab treatment over placebo over the course of treatment (up to 28 months). Notably, in patients without an AFP response, a potentially significant survival benefit was observed for patients treated with ramucirumab compared to placebo (7.2 vs 5.2 months, HR = 0.758, 95% CI 0.600, 0.958; *P* = 0.020), suggesting that even patients with elevated AFP (>1.5 × ULN) who do not have an AFP response may derive a benefit from ramucirumab treatment.

Further analyses showed that 11 patients completely normalised their AFP level, 8 from the ramucirumab arm, and 3 from the placebo arm. The OS for these 11 patients who completely normalised their AFP level was significantly longer than the OS for patients who had AFP response without completely normalising their AFP level (*n* = 111) (25.6 vs 10.6 months, respectively, HR = 0.147, *P* = 0.0019).

## Discussion

Serum AFP has long been recognised as both a diagnostic and prognostic marker.^14–17^ However, assessing AFP kinetics during treatment has been limited. Some retrospective studies have been performed in patients undergoing locoregional therapy, where an AFP response has been associated with a longer survival following transarterial chemoembolisation.^12,18,19^ In the more advanced setting, AFP response has been evaluated in patients being treated with chemotherapy as well as sorafenib where a response is often associated with a survival advantage.^13,20,21^ In these previously published studies, there were fewer than 200 patients evaluated, only one of them derived data from a randomised study and none was placebo-controlled. Here we report post-hoc analyses of AFP response and progression in 565 patients enrolled in the REACH study. Five hundred and sixty patients with assessable changes in AFP were included in our analysis. An additional advantage of having a placebo arm in our REACH study was to allow assessment of AFP kinetics due to underlying HCC rather than treatment-related. However, in most studies examining systemic therapy, including REACH, the number of patients who experience an AFP response has been quite low. Molecularly targeted agents more commonly result in disease stability, and restricting treatment to patients experiencing an AFP response would exclude a large proportion of patients with stable or slowed progression of AFP levels, who would also derive survival benefit from continued treatment.

In the current analysis of REACH, there was an observed benefit with ramucirumab in delaying time to AFP progression, inducing more frequent and deeper AFP response and lesser AFP progression. AFP changes also correlated with radiographic response and progression. The phase 2 biomarker study of ramucirumab as first-line monotherapy in patients with advanced HCC showed that an AFP decrease was more likely in patients who experienced a radiographic response and an AFP increase more likely in patients with radiographically progressive or non-evaluable disease.^22^ Similar correlations have been made between AFP and other radiographic measures of response with other systemic treatments including sorafenib.^13,23–26^ The observations of changes in AFP and measures of objective response in REACH continue to support a correlation between AFP and objective radiographic measures of tumour assessment.

The results presented here support the notion that the ramucirumab antitumour effect is not restricted to patients with AFP or objective tumour response but rather has some activity in all tumours with varying degree. In the ramucirumab arm, more patients experienced both an AFP and a radiographic response compared to placebo. We also observed a shift in the rest of the treated population favouring ramucirumab compared to placebo. More patients in the ramucirumab arm experienced stable AFP or SD compared to placebo. Even in patients who only experienced AFP or radiographic progression, the amplitude of the observed AFP or tumour increase was generally lower.

However, while changes in AFP may correlate with other measures of tumour assessment, neither changes in AFP nor other objective measures of tumour response have been good surrogates to predict OS.^27^ In REACH, while an AFP response was associated with significantly longer OS, analyses support that OS benefit extends to a larger population. Notably, in patients with an elevated baseline AFP (>1.5 × ULN) a potential OS benefit was still observed in ramucirumab-treated patients compared to placebo, even when AFP responders were excluded; this is likely driven by the much larger proportion of patients who experience disease stability rather than regression. While this re-demonstrates that an elevated baseline AFP can identify the subset of patients most likely to derive an OS benefit, the finding also shows that AFP response is inadequate to select patients most likely to derive a survival benefit. Based on the studies presented here, the lack of an AFP response for a patient should not be used in isolation to judge clinical benefit of systemic treatments like ramucirumab.

Of note, a number of patients on placebo also experienced an AFP response. While the reasons for spontaneous AFP response in the placebo arm are unknown, we note that a similar proportion of patients on the  placebo arm also experienced a radiographic response. Other limitations of the results presented here are due to the fact that these were post-hoc analyses performed on a phase 3 study that did not meet its primary end point.

In conclusion, exploratory analyses of REACH show that changes in AFP over time appear to correlate with other measures of objective progression and may help predict patient response, but the utility of AFP to make treatment decisions needs to be validated through a prospective study. Further assessment of the potential benefit of ramucirumab in patients with elevated baseline AFP is being validated in the ongoing REACH-2 study.

## Electronic supplementary material

Supplementary Table S1
