# Supplementary material for: Alpha-fetoprotein kinetics in patients with hepatocellular carcinoma receiving ramucirumab or placebo: an analysis of the phase 3 REACH study
Source: Br J Cancer. 2018 May 29;119(1):19–26. doi: 10.1038/s41416-018-0103-0 (PMC6035236; doi:10.1038/s41416-018-0103-0)
Supplement: Supplementary file 1 — Supplementary Table S1 [file 41416_2018_103_MOESM1_ESM.docx]

Table S1. Demographics and baseline characteristics in patients with levels of AFP ≥ 1.5 ULN

|  |  | **Ramucirumab** | | **Placebo** | |
| --- | --- | --- | --- | --- | --- |
|  |  | ***n* = 205** | | ***n* = 212** | |
| **Age, years** | Median (range) | 63 | (34-87) | 62 | (25-85) |
| **Male** | *n* (%) | 170 | (82.9) | 179 | (84.4) |
| **Race^*^** | *n* (%) |  |  |  |  |
| White |  | 97 | (47.3) | 94 | (44.3) |
| Asian |  | 96 | (46.8) | 110 | (51.9) |
| Black or African American |  | 4 | (2.0) | 3 | (1.4) |
| Other |  | 8 | (3.9) | 5 | (2.4) |
| **ECOG performance status^†^** | *n* (%) |  |  |  |  |
| 0 |  | 112 | (54.6) | 112 | (52.8) |
| 1 |  | 93 | (45.4) | 100 | (47.2) |
| **Geographic region^a^** | *n* (%) |  |  |  |  |
| Region 1: The Americas |  | 19 | (9.3) | 23 | (10.9) |
| Region 2: Europe, Australia and Israel | | 91 | (44.4) | 88 | (41.5) |
| Region 3: Asia |  | 95 | (46.3) | 101 | (47.6) |
| **Etiology of liver disease** | *n* (%) |  |  |  |  |
| Hepatitis B |  | 81 | (39.5) | 83 | (39.2) |
| Hepatitis C |  | 61 | (29.8) | 57 | (26.9) |
| Other |  | 63 | (30.7) | 71 | (33.5) |
| Unknown |  | 22 | (10.7) | 26 | (12.3) |
| Macrovascular invasion present | | 67 | (32.7) | 62 | (29.2) |
| Extrahepatic spread present |  | 150 | (73.2) | 153 | (72.2) |
| **Baseline BCLC stage** | *n* (%) |  |  |  |  |
| Stage B |  | 18 | (8.8) | 24 | (11.3) |
| Stage C |  | 187 | (91.2) | 188 | (88.7) |
| **Prior sorafenib therapy** | *n* (%) |  |  |  |  |
| Sorafenib only |  | 181 | (88.3) | 187 | (88.2) |
| Sorafenib and other systemic therapy | | 24 | (11.7) | 25 | (11.8) |
| **Reason for discontinuation of sorafenib** | *n* (%) |  |  |  |  |
| Progressive disease |  | 180 | (87.8) | 184 | (86.8) |
| Toxicity |  | 25 | (12.2) | 28 | (13.2) |
| **Alpha-fetoprotein** | *n* (%) |  |  |  |  |
| < 400 ng/mL |  | 86 | (42.0) | 81 | (38.2) |
| ≥ 400 ng/mL |  | 119 | (58.0) | 131 | (61.8) |

Abbreviations: BCLC, Barcelona Clinic Liver Cancer; ECOG, Eastern Cooperative Oncology Group; SD, standard deviation; ^*^Race was determined by self-report; ^†^ECOG performance status: 0 indicates asymptomatic, 1 indicates restricted in strenuous activity but ambulatory and able to do light work.

^a^Region 1: Brazil, Canada, U.S.A.; Region 2: Australia, Austria, Belgium, Bulgaria, Czech Republic, Finland, France, Germany, Hungary, Israel, Italy, Netherlands, Norway, Portugal, Romania, Spain, Sweden, Switzerland; Region 3: Hong Kong, Japan, Philippines, South Korea, Taiwan, Thailand
